# Early skin-to-skin contact after cesarean section: A randomized clinical pilot study

**DOI:** 10.1371/journal.pone.0168783

**Published:** 2017-02-23

**Authors:** Martina Kollmann, Lisa Aldrian, Anna Scheuchenegger, Eva Mautner, Sereina A. Herzog, Berndt Urlesberger, Reinhard B. Raggam, Uwe Lang, Barbara Obermayer-Pietsch, Philipp Klaritsch

**Affiliations:** 1 Division of Obstetrics and Maternal Fetal Medicine, Department of Obstetrics and Gynecology, Medical University of Graz, Graz, Austria; 2 Division of Neonatology, Department of Pediatrics and Adolescence Medicine, Medical University of Graz, Graz, Austria; 3 Institute for Medical Informatics, Statistics and Documentation (IMI), Medical University of Graz, Graz, Austria; 4 Clinical Institute of Medical and Chemical Laboratory Diagnostics, Medical University of Graz, Graz, Austria; 5 Division of Angiology, Department of Internal Medicine, Medical University of Graz, Graz, Austria; 6 Division of Endocrinology and Metabolism, Department of Internal Medicine, Medical University of Graz, Graz, Austria; Penang Medical College, MALAYSIA

## Abstract

**Objective:**

Early bonding by skin-to-skin contact (SSC) has been demonstrated to be beneficial for mothers and newborns following vaginal delivery. The aim of this study was to investigate the impact of intraoperative bonding (early SSC) after cesarean section on neonatal adaptation, maternal pain and stress response.

**Study design:**

This prospective, randomized-controlled pilot study was performed at a single academic tertiary hospital (Department of Obstetrics and Gynecology, Medical University of Graz, Austria) between September 2013 and January 2014. Women were randomly assigned to intraoperative (“early”) SCC (n = 17) versus postoperative (“late”) SCC (n = 18). Main variables investigated were neonatal transition (Apgar score, arterial oxygen saturation, heart rate and temperature), maternal pain perception and both maternal and neonatal stress response by measuring the stress biomarkers salivary free cortisol and salivary alpha amylase.

**Results:**

There was no evidence for differences in parameters reflecting neonatal transition or stress response between the ‘Early SSC Group’ and the ‘Late SSC Group’. Maternal salivary cortisol and alpha-amylase levels as well as maternal wellbeing and pain did not differ between the groups. However, the rise of maternal salivary alpha-amylase directly after delivery was higher in the ‘Early SSC Group’ compared to the ‘Late SSC Group’ (p = 0.004).

**Conclusions:**

This study did not reveal significant risks for the newborn in terms of neonatal transition when early SSC is applied in the operating room. Maternal condition and stress marker levels did not differ either, although the rise of maternal salivary alpha-amylase directly after delivery was higher in the ‘Early SSC Group’ compared to the ‘Late SSC Group’, which may indicate a stressor sign due to intensive activation of the sympathetic-adreno-medullary-system. This needs to be further evaluated in a larger prospective randomized trial.

**Trial registration:**

ClinicalTrials.gov NCT01894880

## Introduction

Bonding describes the process by which individuals become emotionally attached to one another. Bonding between mother and child is mainly achieved by early skin-to-skin contact (SSC) starting ideally straight after birth, thus representing the normal behaviour from an evolutionary perspective[1]. However, medical or local conditions may hinder this practice leading to delayed initiation of SSC, which is particularly the case for cesarean deliveries[1–4]. Possible concerns to implement early bonding in the operating room are, beside organizational ones, related to the special environment in the operating room. To promote SSC after cesarean section the Pan American Health Organization and the World Health Organization published a report with suggestions[5]. Some evidence is available regarding SSC after cesarean section[6–12]. However, only four research groups report on immediate SSC in the operating theatre [7, 8, 10–12]. Although there is evidence that SSC is beneficial for neonatal thermoregulation after vaginal delivery, there is few data regarding neonatal thermoregulation after cesarean section [10, 12–17]. Two randomized controlled trials (RCTs) investigated the effect of SSC on infants’ temperatures and found controversial results [10, 12]. One of the latter studies found that the mean neonatal core temperature decreases without active warming from beginning of the surgical procedure until the end of the bonding period. Active skin-surface warming decreases the incidence of hypothermia [10]. However, the second study found that neonates were not prone to hypothermia [12].

Overall there is limited data from RCTs regarding neonatal adaptation after immediate SSC in the operating room. Cesarean delivery disrupts natural childbirth and commonly results in mother-child separation. This may be associated with altered response of infants and mothers to pain and anxiety, thus increasing stress [4, 6].

Several studies have demonstrated an increased secretion of salivary stress biomarkers like the salivary alpha-amylase (sAA) and salivary cortisol (sC) under different stress conditions [18–22]. In addition, investigation of stress biomarkers in saliva has achieved recognition because sample collection is standardized, non-invasive and easy to handle, allowing reflection of direct response to stress since biomarkers are not bound to plasma transport proteins [22–24].

We aimed to investigate the effect of early SSC after cesarean section on neonatal adaptation as well as on maternal pain and stress response.

## Materials and methods

### Study design

This prospective, randomized-controlled pilot study was performed at a single academic tertiary hospital (Department of Obstetrics and Gynecology, Medical University of Graz, Austria) between September 2013 and January 2014. The study was registered with ClinicalTrials.gov (NCT01894880) and approved by the institutional review board (Ethics committee at the Medical University of Graz, Austria; 25–425 ex 12/13). Participants provided their written informed consent to participate in this study.

### Participants

Patients were invited to participate if they were 18 years or older and presented with an indication for elective cesarean section (primary or first repeat cesarean section) at ≥ 37 weeks of gestation. Only singleton pregnancies were included. Patients with suspected abnormal placentation (placenta accreta, increta, or percreta), placenta previa, previous vertical uterine incision, a history of major abdominal surgery, or known fetal malformations were excluded. Eligible patients were approached by members of the research team when they arrived at the delivery suite approximately 2 hours before the scheduled intervention. Informed consent was obtained from all participants.

### Randomization

Immediately before entering the operating room, patients were randomly assigned either to the ‘Early SSC Group’ or the ‘Late SSC Group’ in a ratio of 1:1. Patients and surgeons were informed about randomization results when entering the operating theatre, i.e. no blinding. For allocation of the participants, we used a computer-generated randomization sequence implemented in the Randomizer for Clinical Trials® (www.randomizer.at; Institute for Medical Informatics, Statistics and Documentation, Medical University of Graz, Austria). Clinicians were unaware of the randomization method: block randomization of size six.

### Interventions

Patients arrived at the delivery suite approximately 2 hours before the scheduled intervention. After informed consent was obtained, a venous cannula was inserted for routine purposes and the preoperative blood draw, and a cotton sling which was purpose designed for kangaroo care (BondingTop®, Hoppediz, Bergisch Gladbach, Germany), was attached. Before entering the operating room patients were informed about the group assigned. Surgeries were conducted by the regular staff under spinal anesthesia. In all patients, the umbilical cord was clamped around 10 seconds after delivery and Carbetocin (Pabal®, Ferring Pharmaceuticals, Vienna, Austria) was administered for prevention of post-partum hemorrhage. The newborns were presented to the mothers for a moment before they were transferred to the adjacent neonatology ward to undergo a basic examination. In the ‘Early SSC’ group the babies were brought back to the operating room within 5 minutes to be placed on the mother’s chest and covered by the cotton sling and a cap. SSC was performed continuously until the mother and the newborn were brought back to their room at the delivery suite. In the ‘Late SSC Group’ the babies stayed at the neonatal ward until the end of the surgery. They were handed over to the mothers when they returned to the delivery suite.

### Outcome measures

#### Neonatal transition

Arterial oxygen saturation (SaO2) and heart rate (HR) were measured with the IntelliVue MP50 Monitor (Philips, Eindhoven, Netherlands) and the IntelliVui X2 Monitor (Philipps, Eindhoven, Netherlands). The transducer was immediately placed on the right hand/wrist. Both parameters were recorded every 12 seconds for 25 minutes. Rectal temperatures were measured in the first and the 25^th^ minute of life. Apgar 1, Apgar 5, and Apgar 10 scores were recorded.

#### Maternal and infant salivary stress biomarker

**Fig 1:**
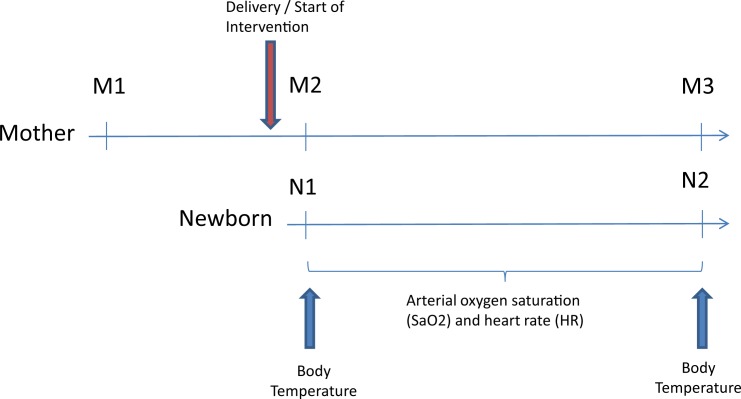
Timing of interventions and measurements (M). The first sample from the mother (M1) was taken 2 hours before the scheduled intervention. The second maternal sample (M2) and the first sample from the newborn (N1) were taken within the first 5 minutes after birth whereas the third maternal (M3) and the second neonatal sample (N2) were taken 25 minutes after birth.

Salivary samples were collected using Salivette® (Sarstedt, Nümbrecht, Germany) according to the manufacturer’s instructions. Salivary samples were obtained from the mother before entering the operating room (Sample Mother 1 [M1]) and from both, mother and child, within the first 5 minutes after delivery (Sample Mother 2 [M2] and Sample Neonate 1 [N1]) as well as 25 minutes after delivery (Sample Mother 3 [M3] and Sample Neonate 2 [N2]). Saliva samples were directly stored in batches at -18°C until performing analysis. Prior to the analysis, saliva samples were thawed and centrifuged for 3 min at 3,300 rpm and then diluted 1:100 with distilled water. The determination of sAA was performed on a Cobas 6000 laboratory analyzer (Roche Diagnostics, Rotkreuz, Switzerland) applying the corresponding alpha-amylase kit (Roche Diagnostics, Rotkreuz, Switzerland), sAA activity was expressed as units per liter (U/l) of saliva. Determination of sC was performed using a fully-automated ELISA (Dynex Technologies, Denkendorf, Germany); values were expressed in ng/mL. All samples were analyzed in duplicates for sC and sAA. (Fig 1)

#### Maternal wellbeing and pain

Intraoperative nausea and vomiting as well as maximum pain (peak pain) on postoperative day (POD)1 and POD2 were evaluated by numeric rating scales (NRS) ranging from 0 to 10, with “0” corresponding to the best result and “10” to the worst one. Nurses documented the level of perceived pain every 8 hours until discharge. On POD1 and POD2 maintenance therapy with intravenous non-steroidal anti-inflammatory drugs (diclofenac, 75 mg every 12 hours) was offered, followed by oral diclofenac (50 mg every 8 hours) on POD3. Systemic opioid analgesics (piritramide, 15 mg every 6 hours) were offered in case of a pain measurement of ≥ 5 on NRS. Patient-controlled analgesia was not utilized [25].

#### Demographic data

Demographic data were extracted from the local perinatal database (PIA, ViewPoint, GE Healthcare, Solingen, Germany) and the medical documentation system or patient files. Neonatal birth weights and umbilical pH values were determined straight after delivery. For evaluation of acidosis umbilical arterial blood was analyzed (ABL 800 FLEX analyzer, Akandevej, Denmark). Acidosis was defined as a postnatal umbilical artery pH < 7.10 [26, 27].

Maternal oxytocin level, maternal bonding behavior, and maternal post-natal depression were also evaluated and will be analyzed and reported in a separate manuscript.

### Sample size

Since there was no representative data available we considered this study as a pilot study. Therefore, no sample size calculation was performed. We aimed to randomize 35 patients.

### Statistical methods

All analyses were performed on intention-to-treat basis. For categorical variables relative and absolute proportions are reported; continuous variables are expressed as median and interquartile range (IQR). Comparisons between the two groups were done for proportions and categorical variables by using Fisher Exact Test and for continuous variables by using Mann-Whitney U test. If appropriate, comparisons between the groups according to two different time points were analyzed by calculating the relative difference between the time points. The change in temperature was investigated by calculating the slope between the two measurements for each newborn taking into account the differences in time frames between the two measurements. For HR as well as for SaO2, a linear mixed model with a fixed effect for time, a fixed effect for group, a random intercept for patient, and a first-order autoregressive covariance structure was used. We calculated for the analysis the mean values of HR and SaO2 for each minute after birth and excluded from the analysis measurements of the first two minutes and after the 26th minute due to more than 50% missing data. No interaction term between time and group was considered because of the limited number of patients.

All analyses were performed using the statistic software R (version 3.1.2, Vienna, Austria). Taking the number of patients in this pilot study into account, this is an exploratory analysis.

## Results

### Participants

**Fig 2:**
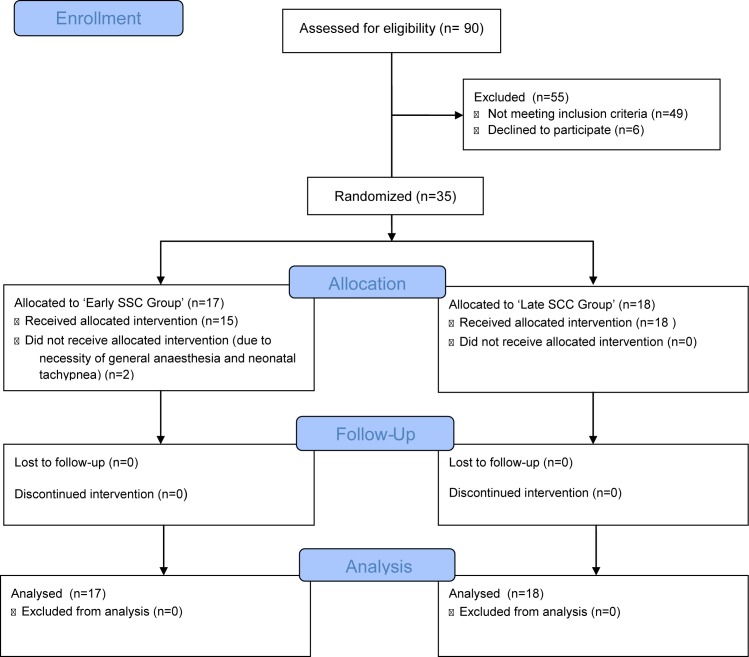
Flow diagram.

A total of 90 pregnant women were assessed for eligibility and 35 were recruited and randomized to either the ‘Early SCC Group’ (n = 17) or the ‘Late SCC Group’ (n = 18) (Fig 2). In two cases from the ‘Early SCC Group’ the intervention was not possible since following randomization general anesthesia was required in one patient and neonatal tachypnea occurred in another.

### Baseline data

Baseline characteristics were similar in the 2 groups for maternal and neonatal parameters (Table 1). Time of sample retrieval did not differ between the ‘Early SSC Group’ and the ‘Late SSC Group’ between the first and the second maternal measurement (p = 0.651) and the second and third maternal measurement and first and second neonatal measurement (p = 0.333) (Table 1).

**Table 1:** Comparison of demographics.

	Early SCC Group n = 17		Late SCC Group n = 18		
	median	IQR	median	IQR	p-value
**Maternal and neonatal characteristics**					
Maternal Age (years)	34	29.5–35.1	32.1	28.7–34.5	0.613
Birthweight (grams)	3,440	3,300–3,720	3,475	3,062–3,655	0.509
Birth length (centimeters)	51	50–53	51	48.2–52	0.433
UA-pH	7.31	7.28–7.32	7.29	7.27–7.30	0.155
UV-pH	7.36	7.35–7.37	7.35	7.34–7.37	0.277
BE	-0.2	-1.8–0.4	-0.2	-1.1–0.6	0.727
**Time of sample retrieval**					
Difference M1 to M2 (minutes)	101.5	63.8–137.5	75	60–132.5	0.651
Difference M2/N1 to M3/N2 (minutes)	24	22–25	25	24–25	0.333

IQR = interquartile range; SCC = skin-to-skin contact; UA = umbilical artery; UV = umbilical vein; BE = base excess; M = maternal measurements (M1 = first [taken before entering the operating room], M2 = second [taken within the first 5 minutes after delivery], M3 = third [taken 25 minutes after delivery]); N = neonatal measurements (N1 = first [taken within the first 5 minutes after delivery], N2 = second [taken 25 minutes after delivery])

### Main results

#### Neonatal transition

**Fig 3:**
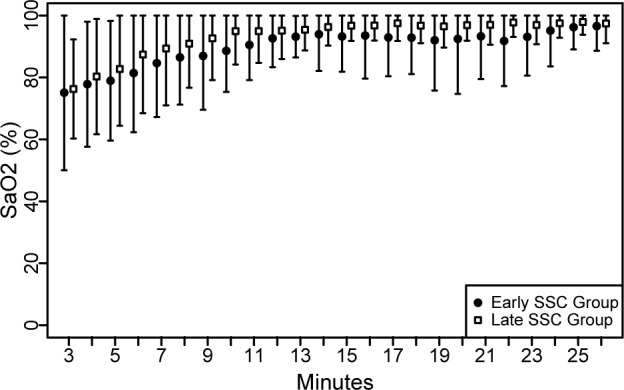
Arterial oxygen saturation (SaO2) of newborns.

Arterial oxygen saturation (SaO2): The linear mixed model showed a dependency between time and SaO2 (p<0.001) but there was no evidence that the SaO2 differs between the groups (p = 0.699) (Fig 3). In the ‘Late SSC Group’, 5 (27.8%) of 18 infants and in the ‘Early SSC Group’ one (5.9%) of 17 infant required supplemental oxygen (p = 0.177). SaO2 results between the two groups remained unchanged after the exclusion of those infants.

**Fig 4:**
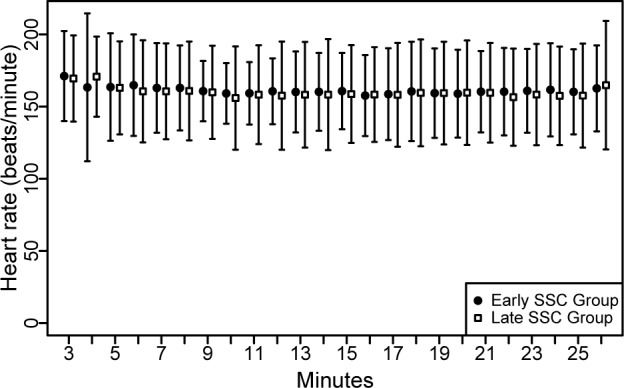
Heart rate (HR) of newborns.

Heart rate (HR): The linear mixed model showed a dependency between time and HR (p = 0.027) but there was no evidence that HR differs between the groups (p = 0.762) (Fig 4).

**Fig 5:**
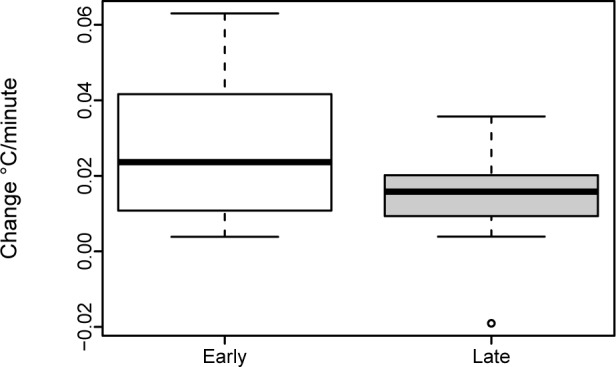
Change in rectal temperatures of newborns.

Temperature: Rectal temperatures were measured in the first minute of live and after 25 minutes. Measurements were available for 88.3% (15/17) of infants in the ‘Early SSC Group’ and all infants in the ‘Late SSC Group’. Rectal temperature was within the normal range in the first minute of life (37.3°C IQR 37.2–37.5°C vs. 37.4°C IQR 37.1–37.6°C) and after 25 minutes (36.7°C IQR 36.5–36.9°C vs. 37.0°C IQR 36.8–37.2°C). Temperature gradient between the two measurements did not differ within the two groups (p = 0.104) (Fig 5).

Apgar: Apgar scores were available for all newborns. 88.3% (15/17) of infants in the ‘Early SSC Group’ and 72.2% (13/18) of infants in the ‘Late SSC Group’ had a one-minute-score of 9, a five-minute-score of 10, and a ten-minute score of 10 (S1 Fig).

#### Maternal and infant salivary stress biomarker

Salivary free Cortisol (sC): Sample M1 was available in 88.3% (15/17) of mothers in the ‘Early SSC Group’ and 100% of mothers in the ‘Late SSC Group’. Sample M2/N1 was obtained in 82.4% (14/17) of mothers and in 64.7% (11/17) of infants in the ‘Early SSC Group’, and 94.4% (17/18) of mothers and 61.1% (11/18) of infants in the ‘Late SSC Group’. Sample M3/N2 could be achieved in 94.1% (16/17) of mothers and 35.3% (6/17) of infants in the ‘Early SSC Group’, and 100% of mothers and 66.7% (12/18) of infants in the ‘Late SSC Group’.

**Fig 6:**
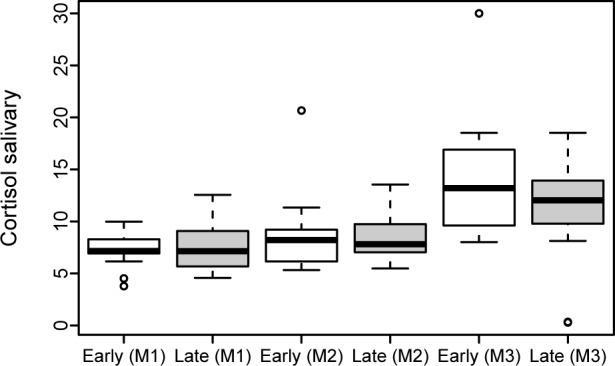
Maternal salivary cortisol (sC) levels.

Maternal sC levels were comparable between the ‘Early SSC Group’ and the ‘Late SSC Group’ at each time point (Table 2). Values increased continuously in both groups from Sample 1 to 3 (Fig 6). The sC gradient from M1 to M2 (p = 0.860) and M2 to M3 (p = 0.281) did not differ between the two groups (S2 Fig).

**Table 2:** Comparison of salivary Cortisol (sC) and salivary alpha-amylase (sAA) levels.

	Early SCC Group n = 17		Late SCC Group n = 18		
	median	IQR	median	IQR	p-value
**Maternal Cortisol Levels**					
M1 (ng/mL)	7.16	6.92–8.29	7.14	5.71–8.76	0.936
M2 (ng/mL)	8.21	6.22–9.16	7.81	7.04–9.74	0.860
M3 (ng/mL)	13.2	9.62–16.35	12.04	9.83–13.82	0.398
**Newborn Cortisol Levels**					
N1 (ng/mL)	14.8	14.2–23.3	18.3	15.1–25.7	NA
N2 (ng/mL)	22.8	17.7–25.2	26.4	19–30	NA
**Maternal Alpha-amylase Levels**					
M1 (U/l)	2,175	1,087–2,914	3,974	1,871–5,172	0.104
M2 (U/l)	5,879	2,934–10,680	4,944	1,902–7,039	0.237
M3 (U/l)	4,676	1,566–10,610	4,965	2,202–6,088	0.618
**Newborn Alpha-amylase Levels**					
N1 (U/l)	238	103–355	359,5	308,2–412,2	NA
N2 (U/l)	391	282,5–499,5	349	329,5–561	NA

IQR = interquartile range; SCC = skin-to-skin contact; M = measurement Mother (M1 = first [taken before entering the operating room], M2 = second [taken within the first 5 minutes after delivery], M3 = third [taken 25 minutes after delivery]); N = measurement Neonate (N1 = first [taken within the first 5 minutes after delivery], N2 = second [taken 25 minutes after delivery]); NA = not available

Due to the high proportion of missing newborn samples, a statistical comparison between the ‘Early SSC Group’ and the ‘Late SSC Group’ was not performed.

The comparison between mothers and newborns in the first minutes (M2 mother and N1 newborn) after delivery showed similar values between the ‘Early SSC Group’ and the ‘Late SSC Group’ (p = 1.0).

Salivary alpha-amylase activity (sAA): Sample M1 was sufficiently available in 88.2% (15/17) of mothers in the ‘Early SSC Group’ and 100% of mothers in the ‘Late SSC Group’. Sample M2/N1 was available in 70.6% (12/17) of mothers and 35.3% (6/17) of infants in the ‘Early SSC Group’, and 77.8% (14/18) of mothers and 33.3% (6/18) of infants in the ‘Late SSC Group’. Sample M3/N2 was available in 58.8% (10/17) of mothers and 11.8% (2/17) of infants in the ‘Early SSC Group’, and 83.3% of mothers and 38.9% (15/18) of infants in the ‘Late SSC Group’.

**Fig 7:**
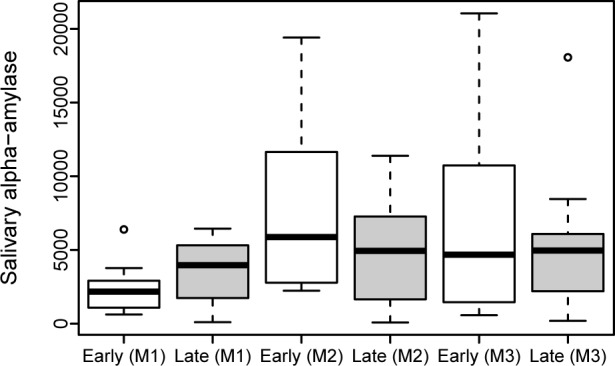
Maternal salivary alpha-amylase (sAA) levels.

Maternal sAA levels were comparable between the ‘Early SSC Group’ and the ‘Late SSC Group’ at each time point (Table 2). Highest values were found directly after delivery. The rise of sAA from M1 to M2 was higher in the ‘Early SSC Group’ (p = 0.004), but did not differ between M2 and M3 (p = 0.471) (Fig 7 and S3 Fig).

The proportion of available newborn samples was too low to do any statistical comparisons between the ‘Early SSC Group’ and the ‘Late SSC Group’.

The comparison between mothers and newborns in the first minutes (M2 mother and N1 newborn) after delivery showed similar values between the ‘Early SSC Group’ and the ‘Late SSC Group’ (p = 0.602).

#### Maternal wellbeing and pain

Maternal pain: Peak pain at rest on POD1 (p = 0.317) and POD2 (p = 0.343) did not differ between the ‘Early SSC Group’ and the ‘Late SSC Group’. Demand for analgesia did not differ between the groups on POD1 (p = 0.419) and POD2 (p = 0.683) (S4 Fig).

Nausea and vomiting: Intraoperative nausea or vomiting was reported by 57.1% (8/14) of women in the Early SSC Group’ and 70.6% (12/17) of women in the ‘Late SSC Group’. The comparison revealed no evidence for a difference (p = 0.442).

### Comment

#### Key results

This prospective, randomized-controlled pilot study found that neonatal transition, salivary free cortisol levels and maternal wellbeing and pain did not differ between the ‘Early SSC Group’ and the ‘Late SSC Group’. Maternal salivary alpha-amylase activity levels increased higher in the first minutes after delivery in the ‘Early SSC Group’ than in the ‘Late SSC Group’.

#### Limitations

Limitations of this study are the limited number of participants and the high proportion of missing data regarding neonatal salivary probes.

#### Interpretation

Our RCT determined neonatal adaptation after early SSC in the operating room and found that all investigated neonatal parameters (arterial oxygen saturation, heart rate, temperature, and Apgar-scores) were comparable with late SCC. Available data on early SSC and newborn physiological behavior following cesarean section are sparse. To the best of our knowledge, there are only two studies on this matter and the earliest initiation of SSC in one of those studies performed by Gouchon et al. was 41 minutes after the cesarean section [6, 9]. As mentioned in the introduction section, there is evidence that SSC significantly supports neonatal thermoregulation after vaginal delivery [17]. However, little is known about the influence of environmental factors in the operating room on the newborns. Studies suggest that newborns delivered via cesarean section are at higher risk for hypothermia. Possible reasons may be the lower temperature in the operating room or maternal locoregional anesthesia [28, 29]. Our study demonstrates that neonatal rectal temperature was within the normal range in both groups and temperature gradient between the two measurements did not differ. These results are in accordance with the results by Beiranvand et al [12]. However, an active skin-surface warming may still be beneficial and supportive under certain circumstances and especially when stable maternal and room temperature cannot be granted [10].

Transient oxygen desaturation is a common physiological process after birth. It may take more than five minutes to reach an arterial oxygen saturation >80% and nearly 10 minutes to reach a saturation >90% [30]. In our population we found that respiratory transition was within the normal range from the start in both groups and did not differ between the groups. However, lower arterial oxygen saturation values are reported to be more frequent after elective cesarean section than vaginal delivery. Similarly, fetal heart rate is also reported to rise slower in infants born via cesarean section and in those born to mothers who received anesthetics (regional epidural/spinal or general) or narcotics during labor [30–35]. Although our findings are reassuring, we suggest monitoring the arterial oxygen saturation and heart rate when early SSC is performed.

Two main physiological stress axes of mothers and their offspring were investigated [22, 36]. Cortisol is essential in the regulation of stress responsiveness and has been used as a measure of the hypothalamic-pituitary-adrenal axis [37–39]. Peak effects on cortisol are usually found to occur 30 minutes after stimulation [40]. Salivary alpha-amylase is mainly secreted by the parotid glands and serves as a measure of the sympathetic-adreno-medullary system [19]. It is reported to increase promptly in response to stressful procedures, such as parachute jump [41]. We found that sC levels did not differ between the ‘Early SSC Group’ and the ‘Late SSC Group’ and that they increased continuously in both groups from Sample 1 to Sample 3. Peak values were found in Sample 3, which is in accordance to previous observations due to a 30 min delay of response [40]. Interestingly, the rise of maternal sAA from M1 to M2 was higher in the ‘Early SSC Group’ compared to the ‘Late SSC Group’. This finding suggests that early SSC additionally triggers the maternal sympathetic-adreno-medullary system. It can be speculated, that this effect may be considered as a stressor due to the immediate contact of the mother with the baby. However, this finding in general and whether this effect is a ‘positive stressor’ or a ‘negative stressor’ would need to be confirmed in larger study populations. To determine whether this is a ‘positive stressor’ or a ‘negative stressor’, the additional performance of a psychological questionnaire might be useful. The collection of salivary samples was quite easy to handle and was well tolerated, although amounts of saliva collected from newborns were limited due to a lesser capacity of saliva production.

Studies already evaluated the implementation of immediate or early SSC in the operating theatre [42, 43]. In our opinion a multidisciplinary team approach is the key to implement early SSC after cesarean section. Changing roles and giving up traditional rituals may be the biggest obstacle to implementation.

### Conclusions

Taking the number of patients into account this study showed that early SSC after cesarean section was not associated with disadvantages for neonatal transition. The rise of maternal sAA directly after delivery was higher in the ‘Early SSC Group’ compared to the ‘Late SSC Group’ which may indicate a stressor sign due to intensive activation of the sympathetic-adreno-medullary-system when early skin contact between mother and newborn is offered. This needs to be further evaluated in a larger prospective randomized trial.

## Supporting information

S1 Checklist CONSORT Checklist. (DOC)

S1 Fig Apgar Scores. Apgar scores after one minute, five minutes and ten minutes.
(EPS)

S2 Fig Maternal salivary cortisol (sC) gradient. Maternal salivary cortisol (sC) gradient between measurements (Panel A: sC gradient from M1 to M2; Panel B: sC gradient from M2 to M3).
(EPS)

S3 Fig Maternal salivary alpha-amylase (sAA) gradient. Maternal salivary alpha-amylase (sAA) gradient between measurements (Panel A: sAA gradient from M1 to M2; Panel B: sAA gradient from M2 to M3).
(EPS)

S4 Fig Peak pain. Peak pain on day 1 and day 2.
(EPS)

S1 Protocol Study Protocol. (PDF)
