# Supplementary material for: Early skin-to-skin contact after cesarean section: A randomized clinical pilot study
Source: PLoS One. 2017 Feb 23;12(2):e0168783. doi: 10.1371/journal.pone.0168783 (PMC5322896; doi:10.1371/journal.pone.0168783)
Supplement: S1 Protocol — (PDF) [file pone.0168783.s006.pdf]

"Pilotstudy: Sectio bonding/Early skin-to-skin contact (SSC) after caesarean section"

date: june 18th, 2013

investigator:

Dr. Martina Kollmann
Universitätsklinik für Frauenheilkunde und Geburtshilfe
Medizinische Universität Graz
Auenbruggerplatz 14
8036 Graz
Tel.: +43/316/385-84190
martina.kollmann@medunigraz.at

Prof. Dr. Philipp Klaritsch
Universitätsklinik für Frauenheilkunde und Geburtshilfe
Medizinische Universität Graz
Auenbruggerplatz 14
8036 Graz
Tel.: +43/316/385-81641
philipp.klaritsch@medunigraz.at

coworkers:

Dr. Anna Scheuchenegger
Doktoratsstudentin der Medizinischen Universität Graz
Tel.: +43/316/385-84547
anna.scheuchenegger@gmail.com

Univ.-Prof. Dr. Berndt Urlesberger
Universitätsklinik für Kinder- und Jugendheilkunde
Medizinische Universität Graz
Auenbruggerplatz 30
8036 Graz
Tel.: +43/316/385-81133
berndt.urlesberger@medunigraz.at

Mag. Dr. Eva Mautner
Universitätsklinik für Frauenheilkunde und Geburtshilfe
Medizinische Universität Graz
Auenbruggerplatz 14
8036 Graz
Tel.: +43/316/38581432
eva.mautner@klinikum-graz.at

Table of contents

1 Background ... 3
2 Hypothesis ... 3
3 Patients ... 3
4 Outcome ... 4
5 Methods ... 6
6 Statistics ... 6
7 Data protection ... 6
8 Risk-benefit analysis ... 6
9 References ... 7

1 Background

In the past decades frequency of cesarean section was increasing in Austria and outside Austria. A retrospective analysis in our institution showed an increase of cesarean sections from 24.2% to 31.9% between 2003 and 2012. Bonding or early skin-to-skin contact (SSC) starts ideally straight after birth[1]. After vaginal delivery bonding/early SSC is already well-established. After cesarean section this important process starts after termination of operation. A Cochran review analyzed randomized studies and shows positive effects of early SSC[1, 2]. Possible concerns to adopt bonding in the operating room are beside organizational ones (change of established processes) also a different ambiance in the operating room (temperature, light, noise). In a prospective randomized trial, early SSC after cesarean section should be analyzed.

Relevance

Early SSC has a positive effect on mothers and newborns. The process of early SSC, which is already establised after vaginal delivery should be implemented and evaluated after cesarean section.

2 Hypothesis

Mothers, who have the chance to bond immediately after birth in the operating room, have lower cortisol, chromogranin A and alpha amylase levels as well as higher oxytocin levels. Adaptation of the newborn is within the normal range. Early bonding has a further positive effect on breast feeding, maternal pain processing and mental health.

3 Patients

Recruitment:

Recruitment will take place in the outpatient department and on the delivery ward after informed consent was obtained.

Inclusion Criteria:

- Age > 18 years
- signed written informed consent
- single pregnancy
- elective cesarean section (between 7am and 15pm)
- Bleeding prophylaxis with Pabal® (carbetocin)

Exclusion Criteria:

- Age < 18 years
- disabled to give signed written informed consent
- Disease of mother or unborn, which could influence the study or makes the study impossible (e.g. severe malformation)
- Non elective cesarean
- Desire to wean
- Desire to leave the hospital within 6 hours after birth
- Bleeding prophylaxis with Syntocinon® (oxytocin)

4 Outcome

Maternal Outcome parameters:

- Oxytocin levels (serum)
    - measurement 1: after admission to the delivery wardreißsaal (Serum 4 ml

- measurement 2: after delivery
- measurement 3: 25 minutes after delivery

- Cortisol, Alpha-Amylase (Saliva[3][mother child ratio])
- (Chromogranin A serum)
    - measurement 1: after admission to the delivery ward (Serum 4 ml
    - measurement 2: after delivery
    - measurement 3: 25 minutes after delivery
- Maternal wellbeing and pain after operation (VAS scale/questionaire)
- Evaluation of postnatale depression (Edinburgh postnatal depression scale)
- Evaluation of bonding (Postpartum Bonding Questionnaire)

Newborn Outcome Parameters:

- Cortisol, Alpha-Amylase (Saliva[3] [mother child ratio])
    - measurement 1: after delivery
    - measurement 2:
        - Early-Bonding-groupe: 25minutes after delivery
        - Late-Bonding-groupe: 25 minutes after delivery
- Transition (body temperature, saturation, heart rate)
- Breastfeeding
    - Aditional feeding
    - Child weight
        - Day 1
        - Day 4
- Delivery weight, weight at discharge

5 Methods

Prospective, randomized pilot study,

Randomization will be performed using the „Web-Randomizer" from the Medical Unversitiy of Graz.

Group 1: Cesarean section and early SSC

Group 2 (Control): Cesarean section and standad care

Study design:

Pilotstudy (2 groups / 15 patients each)

6 Statistics

Descriptive and analytic.

7 Data protection

Data will be stored in an anonymized data set. Investigators and coworkers, as well as health authorities will have access to the non anonymized data set. Those persons are bound to professional discretion. Dissemination of data will happen only for statistical purpose. Publications will also be anonymized. Patients will be coded.

8 Risk-benefit analysis

Probable advantages for the newborn: Early eye and body contact with the mother, positive effect on breastfeeding, better transition.

Probable advantages for the mother: No seperation from the newborn, positive effect on breastfeeding, less anxiety, stress and pain.

Actions which are performed within this study can lead to discomfort (e.g. pain after blood draw). Strong discomfort is not expected.

9 Referenzen

1. Moore ER, Anderson GC, Bergman N, Dowswell T. Early skin-to-skin contact for mothers and their healthy newborn infants. Cochrane Database Syst Rev. 2012 May 16 [cited 3/23/2013 11:26:15 AM];5:CD003519.

2. Hung KJ, Berg O. Early skin-to-skin after cesarean to improve breastfeeding. MCN Am J Matern Child Nurs. 2011 Sep-Oct [cited 3/24/2013 5:40:47 PM];36(5):318,24; quiz 325-6.

3. Inder WJ, Dimeski G, Russell A. Measurement of salivary cortisol in 2012 - laboratory techniques and clinical indications. Clin Endocrinol (Oxf). 2012 Nov [cited 3/24/2013 5:58:49 PM];77(5):645-51.

4. Castro M, Elias PC, Martinelli CE,Jr, Antonini SR, Santiago L, Moreira AC. Salivary cortisol as a tool for physiological studies and diagnostic strategies. Braz J Med Biol Res. 2000 Oct [cited 3/24/2013 5:34:26 PM];33(10):1171-5.

5. Klug I, Dressendorfer R, Strasburger C, Kuhl GP, Reiter HL, Reich A, et al. Cortisol and 17-hydroxyprogesterone levels in saliva of healthy neonates: Normative data and relation to body mass index, arterial cord blood ph and time of sampling after birth. Biol Neonate. 2000 Jul [cited 3/24/2013 5:36:52 PM];78(1):22-6.

6. Nolan A, Lawrence C. A pilot study of a nursing intervention protocol to minimize maternal-infant separation after cesarean birth. J Obstet Gynecol Neonatal Nurs. 2009 Jul-Aug [cited 3/24/2013 5:43:04 PM];38(4):430-42.

7. Ng SM, Drury JA, Turner MA, Didi M, Victor S, Newland P, et al. A novel method of collection of saliva for estimation of steroid levels in extremely premature infants. Acta Paediatr. 2013 Jan 4 [cited 3/24/2013 4:50:16 PM].

8. White-Traut RC, Schwertz D, McFarlin B, Kogan J. Salivary cortisol and behavioral state responses of healthy newborn infants to tactile-only and multisensory interventions. J Obstet Gynecol Neonatal Nurs. 2009 Jan-Feb [cited 3/24/2013 5:43:55 PM];38(1):22-34.
